# Associations of bisphenol and phthalate exposure and anti-Müllerian hormone levels in women of reproductive age

**DOI:** 10.1016/j.eclinm.2024.102734

**Published:** 2024-07-17

**Authors:** Sophia M. Blaauwendraad, Ramon H.M. Dykgraaf, Romy Gaillard, Mengling Liu, Joop S. Laven, Vincent W.V. Jaddoe, Leonardo Trasande

**Affiliations:** a The Generation R Study Group, Erasmus Medical Center (MC), University Medical Center, Rotterdam, the Netherlands; b Department of Pediatrics, Erasmus MC, University Medical Center, Rotterdam, the Netherlands; c Department of Obstetrics and Gynecology, Erasmus Medical Centre, Rotterdam, the Netherlands; d Department of Environmental Medicine, New York University School of Medicine, New York, NY, USA; e New York University College of Global Public Health, New York University, New York, NY, USA

**Keywords:** Bisphenols, Phthalates, anti-Müllerian hormone, Ovarian reserve

## Abstract

**Background:**

In women, exposure to endocrine disrupting chemicals might accelerate the depletion of the ovarian reserve and might be associated with accelerative reproductive aging and fertility. We examined the longitudinal associations of exposure to bisphenols and phthalates with anti-Müllerian hormone concentrations.

**Methods:**

Pregnant women of 18 years or older that resided in Rotterdam between 2002 and 2006 were eligible for participation in this longitudinal prospective cohort study. We measured urinary bisphenol and phthalate concentration at three time-points in pregnancy among 1405 women, of whom 1322 women had serum Anti-Müllerian Hormone (AMH) measurements 6 and/or 9 years postpartum. We performed linear regression models to assess the association of urinary bisphenol and phthalate metabolites with AMH after 6 and 9 years, and linear mixed-effect model to assess the association with AMH over time. Models were adjusted for sociodemographic and lifestyle factors.

**Findings:**

In our multivariable linear regression models we observed associations of higher urinary pregnancy-averaged mono-isobutyl phthalate (mIBP), mono-(2-ethyl-5-oxohexyl) phthalate (mEOHP), and monobenzyl phthalate (mBzBP) with lower serum AMH after both 6 and 9 years. However, these associations did not remain after adjustment for multiple testing. No significant associations of bisphenol A with AMH were present in our study sample. In our linear mixed-effects models, higher mIBP, mono-(2-ethyl-5-hydroxyhexyl) phthalate (mEHHP), mEOHP, and mBzBP were associated with lower overall AMH levels (differences −0.07 (95% CI −0.13, −0.02), −0.09 (−0.15, −0.02), −0.08 (95% CI −0.14, −0.02), and −0.08 (−0.13, −0.03) μg/L per doubling in mIBP, mEHHP, mEOHP, and mBzBP respectively) (all False Discovery Rate adjusted p-values < 0.05).

**Interpretation:**

We identify decreases in indices of ovarian reserve in relationship to prenatal phthalate exposures. Studies are needed replicating our results among large multi-ethnic non-pregnant populations and assessing transgenerational effects of exposure on ovarian reserve.

**Funding:**

This study was supported by the 10.13039/501100003061Erasmus Medical Center and 10.13039/501100001828Erasmus University Rotterdam, the 10.13039/501100001826Netherlands Organisation for Health Research and Development, the 10.13039/501100000781European Research Council, the Dutch Heart Foundation, the 10.13039/501100003092Dutch Diabetes Foundation, the European Union’s Horizon 2020 Research and Innovation Program, the 10.13039/100000002National Institutes of Health, Ansh Labs Webster, and the 10.13039/501100001722Royal Netherlands Academy of Arts and Sciences.

**Research in context**

**Evidence before this study**

Bisphenols and phthalates are ubiquitous chemicals that interfere with estrogen and androgen receptors, and have shown to increase oxidative stress, reduce follicle growth, and increase atresia in rodent follicles. In humans, exposure to bisphenols and phthalates might accelerate depletion of the ovarian reserve, of which serum Anti-Müllerian hormone (AMH) has shown to be the best available measure. Studies assessing the association of phthalate and bisphenol exposure with AMH levels in women of reproductive age have predominantly been performed in selective populations of subfertile couples, of which some reported associations of higher urinary bisphenol A (BPA), S (BPS) and mono-(2-5-oxohexyl) phthalate (mEOHP) with lower serum AMH levels. Only one study has been performed in the general population among 297 women of childbearing age, reporting a cross-sectional association of higher mono-isobutyl (mIBP) only with lower serum AMH.

**Added value of this study**

To our best knowledge, we are the first to assess the longitudinal associations of exposure to bisphenols and phthalates with repeated AMH levels over time. Our study adds to the existing literature as it includes a large study sample of 1322 women of childbearing age within the general population. Also, we used repeated measurements of the highly variable bisphenols and phthalates, which causes a more accurate estimation of exposure.

**Implications of all the available evidence**

We identify decreases in indices of ovarian reserve in relationship to prenatal phthalate exposure. The rising global prevalence of infertility requires identification of contributing environmental factors. Therefore, further studies are needed replicating our results among large multi-ethnic non-pregnant populations and assessing transgenerational effects of exposure to bisphenols and phthalates on male and female fertility parameters.

## Introduction

Globally, the prevalence of infertility is increasing in an alarming pace.^1^ In women, several factors have been suggested to speed up the depletion of the ovarian reserve and have been associated with accelerative reproductive aging and fertility.^2^ Anti-Müllerian hormone (AMH) has shown to be the best available measure of ovarian reserve, and its clinical application includes the assessment of ovarian reserve in the clinical diagnosis of several fertility related diseases.^3^ In particular, it is a predictor in the context of response to ovarian stimulation in assisted reproductive technologies. In women of reproductive age, AMH is produced by granulosa cells of ovarian follicles during the early stages of follicle development. AMH regulates folliculogenesis by inhibiting primordial follicle recruitment and decreasing sensitivity of small antral follicles to follicle-stimulating hormone.^4^

Bisphenols and phthalates are ubiquitous endocrine-disrupting chemicals that are widely applied in common consumer products.^5^ Bisphenols are used in the production of polycarbonate plastics and epoxy resins, applied in food can coatings, toys, water pipes and thermal papers. Phthalates are used in industrial manufacturing to supply plastic products with elasticity. Low-molecular-weight (LMW) phthalates are applied in personal care products, solvents, or adhesives. High-molecular-weight (HMW) phthalates are incorporated in vinyl plastics.^6^ In humans, bisphenols and phthalates have been detected in urine, serum, amniotic fluid, and follicular fluid.^2^^,^^7^

Bisphenols and phthalates act as weak ligands capable of interfering with amongst others estrogen and androgen receptors, causing dysregulation of hormonal signaling.^5^ Also, *in vitro* and animal studies have reported that in gonadal cells, bisphenols and phthalates interact via genomic, non-genomic and epigenetic mechanisms to alter gene expression and cell proliferation, and induce apoptosis. In addition, they have shown to induce inflammation and oxidative stress. Through these mechanisms, bisphenols and phthalates exposure might contribute to accelerated reproductive aging.

Previous studies assessing the association of phthalate and bisphenol exposure with AMH levels in women of reproductive age have predominantly been performed in infertility clinics, subsequently within a selective population, and have cross-sectional designs.^8^, ^9^, ^10^, ^11^, ^12^, ^13^ Some, but not all, studies reported associations of higher urinary bisphenol A (BPA), S (BPS) and mono-(2-5-oxohexyl) phthalate (mEOHP) with lower serum AMH levels. One study in the general population among 297 women of childbearing age reported a cross-sectional association of higher mono-isobutyl (mIBP) only with lower serum AMH.^14^ Among women of menopausal age, higher phthalate concentrations have been associated with both higher^15^^,^^16^ and lower^17^ AMH levels. Among 1189 women aged 45–56 years, higher concentrations of di-2-ethylhexyl (DEHP) metabolites were associated with lower serum AMH levels.^17^ Contrary, a study among 718 women aged 45 years old reported that higher concentrations of anti-androgenic phthalates–which included DEHP metabolites, mono-benzyl phthalate (mBzP), mono-butyl phthalate (mBP), and mIBP–were associated with higher AMH.^16^ Similar results on mBzP and mBP were reported in a recently published study among 614 women aged 45–54 years.^15^

To get more insight on the potential longitudinal associations of bisphenol and phthalate exposure on ovarian reserve, studies among larger samples sizes in the general population are needed, including AMH measurements at multiple time points. Therefore, in a population based prospective cohort study among 1322 women, we assessed the association of pregnancy-averaged concentrations of phthalates and bisphenols from three urinary samples with serum AMH concentrations after 6 and 9 years.

## Methods

### Study design and population

**Fig. 1:**
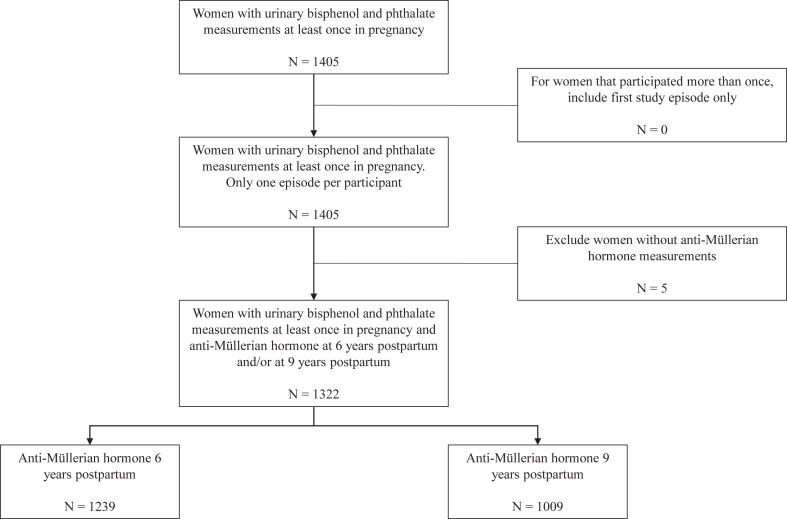
**Flowchart of participants included in the study**.

This study is embedded in the Generation R Study, a population-based prospective cohort study from fetal life until adulthood in Rotterdam, the Netherlands.^18^ The study is designed to identify early environmental and genetic causes and causal pathways leading to adverse health outcomes from fetal life till young adulthood. Pregnant women living in Rotterdam with an expected delivery date between April 2002 and January 2006 were eligible for participation and enrolled in the study. In total, 8879 women were enrolled, of which 76% before a gestational age of 18 weeks. The current study followed the recommendations of the Strengthening the Reporting of Observational studies in Epidemiology (STROBE) checklist. A subsample of 1405 women had bisphenol and phthalate measurement at least once in pregnancy, whom all had only one episode of participation. Of those, 1239 women had AMH measurement at 6 years postpartum, and 1009 at 9 years postpartum follow up (Flowchart shown in Fig. 1).

### Ethics

Study approval was obtained by the Medical Ethical Committee of the Erasmus Medical Center, University Medical Center, Rotterdam (MEC 198.782/2001/31). Written informed consent was obtained from all women.

### Maternal urinary bisphenol and phthalate measurements

Maternal bisphenol and phthalate concentrations were measured in spot urine samples obtained from each woman at three time points during pregnancy (median 12.9 weeks of gestation (interquartile range (IQR) 12.0, 14.4); median 20.4 (IQR 19.9, 20.9); median 30.2 (IQR 28.8, 32.4)). Details of bisphenol, phthalate and creatinine measurements have been described previously.^19^ The absolute mean recoveries of the compounds was acceptable, with a percentage RSD (% RSD) varying from 12.9 to 23.6%, showing acceptable precision and reliability.^19^ Descriptive statistics of the bisphenols and phthalate concentrations in our study sample are shown in Supplementary Table S1. Analyses were performed at the Wadsworth Center, New York State Department of Health, Albany, New York, and at New York School of Medicine, New York, New York, by the same procedures and laboratory personnel.

Chemicals were included for individual analysis and grouping when they were detectable in ≥50% of the samples at all three time points (Supplementary Table S2). Concentrations of exposure were expressed in nanomoles per liter urine (nmol/L). We grouped phthalates according to their molecular weight and parent phthalates into low-molecular-weight (LMW) and high-molecular-weight (HMW) phthalates. Details on chemicals included in grouping per trimester are shown in Supplementary Text S1. Grouping was performed by calculating the weighted molar sums of the individual metabolites. Concentrations below the limit of detection (LOD) were imputed by the LOD/√2, which has shown to provide an accurate estimation of the mean and standard deviation (SD) of exposure values in a left-centered log-normal distribution, also when compared to a maximum likelihood statistical method.^20^ We calculated the mean exposure during pregnancy, to reduce the potential for exposure misclassification due to temporal variability. Urinary bisphenol and phthalate concentrations were log_2_ transformed to account for right skewedness. Therefore, all estimates referring to the chemicals concentrations are expressed per doubling in chemical concentration. To account for urinary dilutions, we adjusted for maternal urinary creatinine concentrations in micrograms per liter in all our models.^21^

### Anti-Müllerian hormonal assays

Maternal serum samples were obtained postpartum at 6 (maternal age 36.8 years (±SD 4.7)) and 9 (maternal age 40.9 years (±SD 4.5)) years follow up, as previously described.^18^^,^^22^ Venous samples were obtained by trained research nurses and stored at room temperature, before being transported to the regional laboratory for processing and storage (STAR-MDC). Processing was planned to finish within 3 h after venous puncture. Samples were centrifuged and stored at −80 °C.

AMH measurements were performed using the AnshLabs pico AMH ELISA (AnshLabs, Webster, TX, USA), according to standard procedures. The samples were thawed and measured on the same day. Loss of signal for AMH due to prolonged storage at −80 °C was deemed negligible given our experience with in-house used quality control materials.^23^ During the study, kit controls as well as pooled serum controls were used to assure accuracy. The ICC between the AMH measurements at the two different time points was 0.76 (95% CI 0.61, 0.84). Values below the limit of detection (0.023 μg/L) were imputed by the LOD/√2.^20^ We calculated the average AMH per person by calculating the sum of the measurements and dividing it by the number of measurement. We calculated the change in AMH by subtraction AMH concentrations at 6 years postpartum from AMH concentration 9 years postpartum. To account for right skewedness, AMH values were log transformed.

### Covariates

We obtained information on maternal age, educational level (completed primary school, secondary school or higher), ethnicity (Dutch, Western, non-Western), parity (nulliparous or parous), alcohol use (yes or no) and smoking in pregnancy (yes or no) through questionnaire. Maternal height was measured at time of enrollment and weight was obtained in early pregnancy, and at the 6 and 9 years postpartum visits. Body mass index (BMI) for all visits was calculated.

### Statistics

First, we assessed the correlation of the chemical concentrations between trimesters using Spearman’s correlation. Second, we assessed the correlation of AMH levels 6 years postpartum and 9 years postpartum using Spearman’s correlation. Third, we performed linear regression to assess the associations pregnancy-averaged concentrations of the exposures (the individual and grouped bisphenols and phthalates) with serum AMH at 6 and 9 years postpartum. Fourth, we assessed the associations of pregnancy-averaged concentrations of the exposure with average AMH and change in AMH between 6 and 9 years postpartum. We checked for the linear regression assumptions of normality of the residuals by visual inspection of QQ plots, linearity by visual inspection plotting the residuals versus the predicted values, independence through Durbin–Watson tests, and homoscedasticity by visual inspection of scale-location plots. As AMH levels are dependent on age, all models were adjusted for maternal age at AMH measurement and urinary creatinine concentrations in the basic model and additionally for sociodemographic and lifestyle factors in the adjusted models. Potential confounders were selected based on literature and depicted in an Directed Acyclic Graph (Supplementary Fig. S1). We included variables that were associated with the exposure and outcome as confounders in our adjusted models. We used ethnicity and educational level as proxy for socio-economic status. Our final confounders were early pregnancy body BMI, education, ethnicity, parity smoking, and alcohol use. As we considered BMI at outcome measurement a potential mediator, we performed a mediation analysis of the adjusted linear regression models between the exposure groups and the outcomes AMH at 6 and 9 years postpartum. We fitted a model for the mediator BMI including all confounders, and a model for the outcomes including all confounders and the mediator BMI. We assessed the natural average causal mediation effects, the natural average direct effects, and the proportion mediated. We obtained the confidence intervals of the causal mediation effects using bootstrapping with 1000 resamplings, summarizing the results using heteroscedasticity-consistent standard errors in the quasi-Bayesian Monte Carlo method based on normal approximation. Next, as ethnicity has shown to play an important role in AMH levels and thus might be considered a potential effect modifier, we repeated our adjusted analysis in women with a Dutch ethnicity only.^24^

As a secondary analysis, we estimated the main association of the exposures with AMH over time. We used linear mixed-effects models to estimate the mean difference in repeated AMH measures for an IQR increase in pregnancy-averaged concentrations of the exposures. The outcome AMH included the AMH measurements at 6 years postpartum and at 9 years postpartum. We included a random subject-specific intercept. We assumed a normal distribution of the random-effects term. We did not include a random slope as this did not improve the model fit using likelihood ratio test. We tested for the assumptions of the linear mixed-effects model. We assessed linearity and constant variance by plotting the residuals versus the fitted values, and normality of the residuals using QQ-plots. We tested for interaction of the exposures with time by adding an interaction term to the models; there was no interaction with time. Basic models were adjusted for urinary creatinine concentrations and maternal age at baseline. Full models were additionally adjusted for early pregnancy body mass index, education, ethnicity, parity smoking, and alcohol use.

Missing values were imputed using multiple imputation by the fully conditional specification method, and pooled results from 25 imputed datasets were reported. Predictors in the model were age of mother at baseline and at 9 years follow-up, and maternal ethnicity, education, parity, smoking and alcohol use in pregnancy, and body mass index at baseline, 5-year follow-up, and 9-year follow-up. The percentage of missing values ranged from 0.0% to 20.1% (largest number of missing values for maternal BMI 9 years postpartum). All statistical tests were 2-sided and p-values for all analysis were presented. Because of the explorative nature of the study, we considered Nominal adjusted p-values < 0.05 significant. Subsequently, to adjust for multiple hypothesis testing, we considered a False Discovery Rate (FDR) adjusted p-value threshold of <0.05. Analyses were performed using R Statistical Software (version 4.3.2; R Development Core Team).

### Role of the funding sources

The funding sources had no role in the study design; in the collection, analysis, or interpretation of data; in the writing of the report; and in the decision to submit the paper for publication.

## Results

### Characteristics

Table 1 presents the characteristics of the study population. The mean maternal age at inclusion was 30.6 years, and at 6 and 9 years follow up 37.2 and 40.9 years, respectively. Most women were Dutch (53.6%), highly educated (50.4%), nulliparous (60.8%), did not smoke but used alcohol in pregnancy. Median serum AMH levels were at 6 years postpartum 1.827 μg/L (0.016, 0.299), and at 9 years postpartum 1.106 μg/L (0.016, 0.028). The Spearman correlations of the maternal urinary chemical concentrations between trimesters was generally low (Supplementary Fig. S2). The Spearman correlation between 6 years and at 9 years AMH was 0.86.

**Table 1:** Characteristics of the study population.

General characteristics	Total (n = 1322)
Age at exposure measurement years, mean (SD)	30.6 (4.8)
Ethnicity, n (%)	
Dutch	714 (54.5)
European	202 (15.4)
Non-European	393 (30.0)
Highest education finished, n (%)	
Primary	94 (7.4)
Secondary	525 (41.5)
Higher	646 (51.1)
Parity, n (%)	
Nullipara	804 (61.2)
Multipara	510 (38.8)
Body mass index early pregnancy in kg/m^2^, median (IQR)	23.4 (21.5, 26.2)
Body mass index 6 years in kg/m^2^, median (IQR)	24.5 (22.1, 28.2)
Body mass index 9 years in kg/m^2^, median (IQR)	24.6 (22.1, 28.3)
Smoking, yes, n (%)	299 (25.0)
Alcohol use, yes, n (%)	691 (58.2)
**Outcome characteristics**	
Anti-Müllerian hormone levels 6 years, μg/L, median (IQR)	1.827 (0.822, 3.541)
Age at measurement 6 years, mean (SD)	37.2 (4.7)
Anti-Müllerian hormone levels 9 years, μg/L, median (IQR)	1.109 (0.293, 2.530)
Age at measurement 9 years, mean (SD)	40.9 (4.5)

Values presented as mean (standard deviation (SD)), median (interquartile range (IQR)) or number of participants (valid %). Missing per covariate, n (%): ethnicity 13 (1.0), education 57 (4.3), parity 8 (0.6), body mass index early pregnancy 6 (0.5), body mass index 6 years 29 (2.2), body mass index 9 years 266 (20.1), smoking 128 (9.7), alcohol 134 (10.1).

### Bisphenols, phthalates, and serum AMH at each time point

**Fig. 2:**
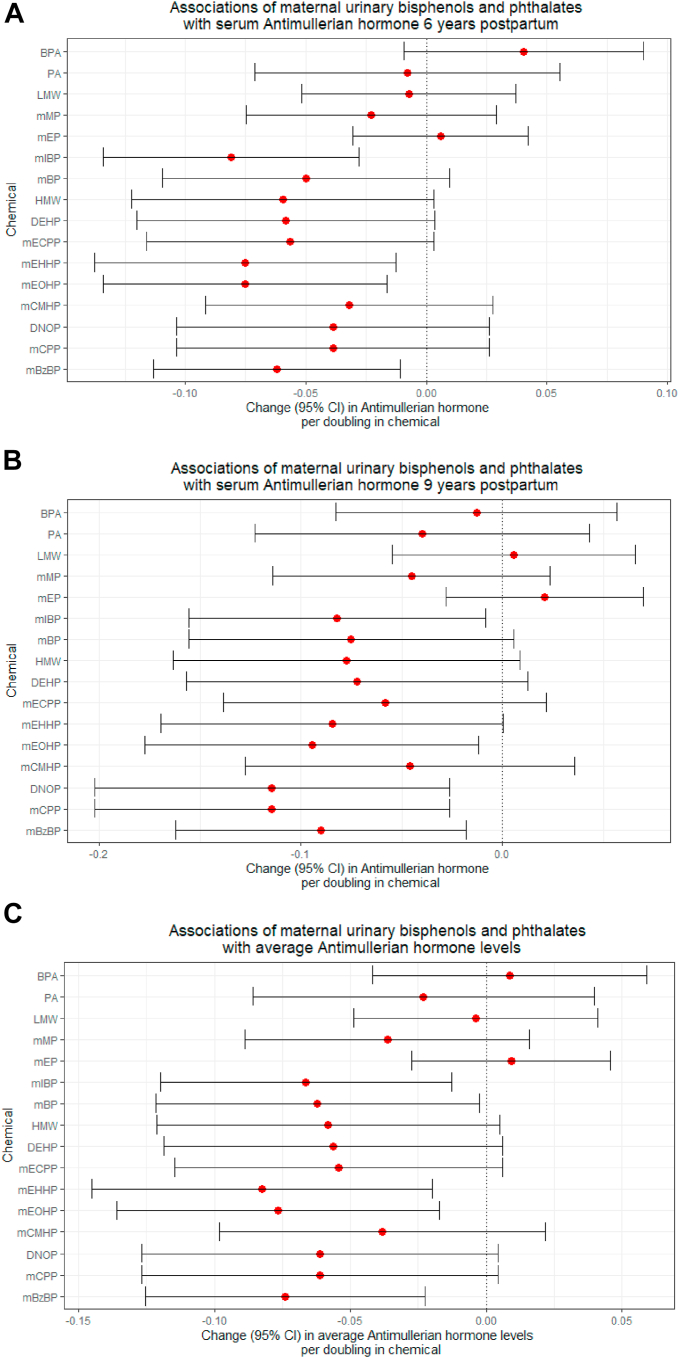
**Associations of urinary bisphenol and phthalate concentrations with serum anti-Müllerian hormone levels in A) 6 years and B) 9 years postpartum, and C) on average, obtained from multivariable linear regression models.** Models reflect the change (95% confidence interval (CI)) in maternal serum Anti-Müllerian hormone (AMH) levels (μg/L) 6 years and 9 years postpartum, and on average per doubling in maternal urinary pregnancy-averaged bisphenol or phthalate metabolite or metabolite group (nmol/L). Models are adjusted for maternal urinary creatinine concentrations, maternal age at exposure and outcome measurement, ethnicity, educational level, parity, smoking, alcohol use and body-mass index at exposure measurement. BPA bisphenol A, BPS bisphenol S, BPF bisphenol F, PA phthalic acid, mMP monomethylphthalate, mEP monoethylphthalate, mIBP mono-isobutylphthalate, mBP mono-n-butylphthalate, mECPP mono-(2-ethyl-5-carboxypentyl)phthalate, mEHHP mono-(2-ethyl-5-hydroxyhexyl)phthalate, mEOHP mono-(2-ethyl-5-oxohexyl)phthalate, mCMHP mono[(2-carboxymethyl)-hexyl]phthalate, mBzP monobenzylphthalate, mHxP mono-hexylphthalate, mHpP mono-2-heptylphthalate, BP total bisphenol, LMW low molecular weight phthalate, DEHP di-2-ethylhexyl phthalate, DNOP di-n-octylphthalate, HMW high molecular weight phthalate. Corresponding data are presented in Supplementary Table S5.

In our multivariable linear regression models, no significant associations of bisphenol A with AMH levels were present. Higher pregnancy-averaged mIBP, mono-(2-ethyl-5-hydoxyhexyl phthalate (mEHHP), mEOHP and mBzBP were associated with lower serum AMH concentrations after 6 years (differences mIBP −0.08 (−0.13, −0.03) μg/L per doubling in exposure; mEHHP −0.08 (−0.14, −0.01); mEOHP −0.08 (−0.13, −0.02); and mBzBP −0.06 (−0.11, −0.01)) (Fig. 2A, Supplementary Tables S4 and S5). Likewise, higher pregnancy-averaged mIBP, mEOHP, mono(3-carboxypropyl) phthalate (mCPP) and mBzBP were associated with lower serum AMH concentrations after 9 years (differences mIBP −0.08 (−0.16, −0.01); mEOHP −0.09 (−0.18, −0.01); mCPP/DNOP −0.11 (−0.20, −0.03); and mBzBP −0.09 (−0.16, −0.02)) (Fig. 2B). Higher pregnancy-averaged mIBP, mBP, mEHHP, mEOHP, and mBzBP were associated with higher average AMH concentration (Fig. 2C). Only the association of higher mIBP with lower AMH 6 years postpartum remained significant with FDR-correction for multiple testing. No significant associations with change in AMH between 6 and 9 years were present in our study sample.

In our mediation analysis, maternal body mass index did not mediate any of the associations of the chemicals with AMH (Supplementary Table S6). Repeating our analysis among Dutch women solely did not change the direction of the associations (Supplementary Table S7).

### Main average associations of bisphenols and phthalates with AMH over time

**Fig. 3:**
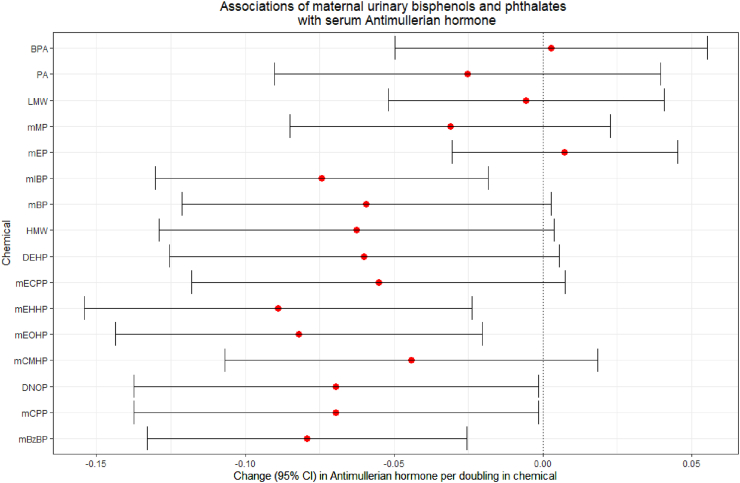
**Associations of urinary bisphenol and phthalate concentrations with serum anti-Müllerian hormone levels, obtained from linear mixed-effects models, adjusted model.** Models reflect the change (95% confidence interval (CI)) in maternal serum Anti-Müllerian (AMH) hormone levels (ug/L) per doubling in maternal urinary pregnancy-averaged bisphenol or phthalate metabolite or metabolite group (nmol/L). Effect estimates were obtained from linear mixed-effects models and adjusted for maternal urinary creatinine concentrations, maternal age at baseline, ethnicity, educational level, parity, smoking, alcohol use and body-mass index at exposure measurement. BPA bisphenol A, BPS bisphenol S, BPF bisphenol F, PA phthalic acid, mMP monomethylphthalate, mEP monoethylphthalate, mIBP mono-isobutylphthalate, mBP mono-n-butylphthalate, mECPP mono-(2-ethyl-5-carboxypentyl)phthalate, mEHHP mono-(2-ethyl-5-hydroxyhexyl)phthalate, mEOHP mono-(2-ethyl-5-oxohexyl)phthalate, mCMHP mono[(2-carboxymethyl)-hexyl]phthalate, mBzP monobenzylphthalate, mHxP mono-hexylphthalate, mHpP mono-2-heptylphthalate, BP total bisphenol, LMW low molecular weight phthalate, DEHP di-2-ethylhexyl phthalate, DNOP di-n-octylphthalate, HMW high molecular weight phthalate. Corresponding data are shown in Supplementary Table S9.

In our linear mixed-effects models, we observed associations of higher maternal urinary pregnancy-averaged mIBP, mEHHP, mEOHP, mCPP, and mBzBP concentrations with lower AMH levels between 6 years and 9 years postpartum (Fig. 3, Supplementary Tables S8 and S9). The strongest associations, that remained after FDR-adjustment for multiple testing, were present for mIBP, mEHHP, mEOHP, and mBzBP (differences mIBP −0.07 (95% CI −0.13, −0.02) μg/L per doubling in exposure; mEHHP −0.09 (−0.15, −0.02); mEOHP −0.08 (95% CI −0.14, −0.02); and mBzBP −0.08 (−0.13, −0.03)).

## Discussion

In this population based study among 1322 women, we reported associations of higher pregnancy-averaged concentrations of several phthalates with lower AMH levels after 6 year and after 9 years, and with overall AMH levels. The strongest concentrations were present for pregnancy-averaged mIBP, mEHHP, mEOHP, and mBzBP.

In women of reproductive age, previous studies on the effect of bisphenol and phthalate exposure on AMH levels are predominantly cross-sectional studies performed in infertility clinics.^8^, ^9^, ^10^, ^11^, ^12^, ^13^ On bisphenols, four studies have been carried out among 100 to 500 women visiting fertility clinics. Two studies in Korea and Poland reported associations of higher spot urine BPA with lower serum AMH.^9^^,^^11^ A Chinese study reported associations of higher BPA with a lower antral follicle count, but not with AMH levels.^13^ Contrary, among Chinese women, higher spot urine BPS, but not BPA or BPF, was associated with lower serum AMH.^10^ On phthalates, one study has been performed among the general population including 297 Chinese women of reproductive age, reporting a negative association of mIBP with AMH.^14^ Other studies in infertility clinics in Korea (307 women), the US (138 women) and China (415 women) assessed several low and high molecular weight phthalates, of which only the US study reported an association of higher mEOHP with lower AMH in follicular fluid.^8^^,^^9^^,^^12^ Among menopausal women, a US study among 1189 women assessing the longitudinal association of phthalate exposure with AMH reported that higher concentrations of mECPP, mEHHP, and mEOHP were associated with lower AMH.^17^ Contrary, in two cross-sectional US studies among 614 and 718 menopausal women, higher mCPP, mBzP, mBP, and/or of the sum of anti-androgenic phthalates were associated with higher AMH.^15^^,^^16^ Inconsistencies between those previous studies could arise from differences in population, as most studies have been performed in infertility clinics or women of menopausal age. Also, none of these studies has investigated long-term associations.

In the current study, we aimed to assess the association of higher exposure to bisphenols and phthalates with AMH levels over time within women of reproductive age in the general population. We observed consistent associations of higher mIBP, mEHHP, mEOHP and mBzBP with lower AMH after 6 years, 9 years, and overall. Studies both among women of reproductive age as well as menopausal women have reported similar associations of the low molecular weight phthalate mIBP^14^ and high molecular weight phthalates mEHHP and mEOHP^8^^,^^17^ with lower AMH. In our study sample, we reported no significant associations with change in AMH between 6 and 9 years postpartum, possibly because associations of the exposures with AMH do not accumulate over time. Although our associations attenuated towards zero with adjustment for multiple hypothesis testing in our linear regression models, associations remained significant in our linear mixed-effects models. In our study, linear-mixed effects models might be particularly suitable, as they can test the overall associations of exposures with repeated outcome measures and can easily handle missing values. Thus, our results suggest an effect of exposure to several phthalates with AMH levels over time in women of reproductive age. Our results are hypothesis generating and of interest for future studies. Although effect estimates were small, they are of importance on a population health level. From a clinical perspective, changes of 0.1 or 0.2 μg/L serum AMH might have particularly great impact in women that have low AMH values, around the 5th percentile of the population, whereas impact in women in the higher AMH percentiles might be less significant. Additionally, the concentrations of bisphenols and phthalates observed in our study cohort are similar to those observed in other Western studies from the same time period, justifying extrapolation to the general population.^19^

Plausible mechanisms of action of phthalate-induced disruption of the ovaries might through oxidative stress and disruption of gene expression through epigenetic mechanisms.^2^ DNA repair and maintenances genes are heavily involved in the age at which menopause will ensure.^25^ The majority of these genes are involved in double strand break repair, and in case women are equipped with the least effective genes, they have a 4–5 fold increased risk on entering the menopause before age 45 years and on premature ovarian failure.^25^ Most of double strand breaks result from exposure to reactive oxygen species and other radicals. As phthalates have shown to increase oxidative stress markers, they might cause double strand breaks and decrease the age at which menopause will ensure, which will be reflected in lower AMH levels.^26^^,^^27^ This is supported by *in vitro* and *in vivo* studies in rodents on the effects of exposure to several individual phthalates and mixtures on follicles, that have been conducted over a wide range of phthalate concentrations. Collectively, these studies found that increased oxidative stress, reduced follicle growth, and increased atresia. Another mechanisms of effect is through systemic endocrine disruption, as bisphenols and phthalates interfere with amongst others estrogen and androgen receptors, causing dysregulation of hormonal signaling. An imbalance in hormonal assets, required for the preservation and maturation of ovarian follicles, can lead to their premature aging or death.^28^ Differences in the effects of bisphenols and phthalates on maternal AMH levels might be explained by their endocrine disrupting capacities. Bisphenols have stronger estrogenic capacities, whereas phthalates have stronger anti-androgenic effects. In the ovaries, the regulation of follicular atresia involves a balance of pro- and anti-apoptotic factors. Interestingly, estrogens help prevent follicles from undergoing atresia, whereas androgens promote apoptosis and ultimately atresia.^29^ Additionally, *in vivo* studies have demonstrated that DEHP, the parent phthalate of mEHHP and mEOHP, inhibits antral follicle growth through oxidative stress.^29^ Increased apoptosis and diminished ovarian reserve might subsequently be reflected in decreased AMH levels.^4^^,^^30^

The current study benefitted from the large multi-ethnic study population, the prospective data collection from pregnancy onwards, and the availability of a wide range of covariates. Bisphenols and phthalates are generally lipophilic, have short biological half-lives of less than 24 h, and undergo a first-pass effect when ingested orally before excretion in urine.^31^ Although bisphenols and phthalates can also be measured in blood, urine is often used as it is noninvasive and notwithstanding the short biological half-life, it might reasonably reflect exposure up to 3 months or even longer, as sources of exposure remain constant.^32^ However, within-person variability of urinary bisphenol is reported to be high, which might have caused attenuation bias, despite the use of repeated measurements in our study. Urinary samples were stored at −20 °C for approximately 10 years. Studies suggest that −80 °C would be a more optimal storage temperature, but no studies investigated differences between these temperatures and effects of long-term storage.^33^ Therefore, we cannot exclude biological activity during the storage period leading to potential non-differential misclassification and underestimation of the effects. The lack of international standards on AMH measurements make comparability of values impossible. Serum AMH concentrations show minor fluctuations over short time spans and are therefore representative measures in non-pregnant women.^24^ Although AMH is quite constant throughout the menstrual cycle, it might be lower in the mid menstrual cycle range, which is more pronounced in older women. Also, AMH levels might be influenced by the use of hormonal contraceptives. Nevertheless, AMH is produced as early on in the smallest developing follicles which have just escaped the primordial follicle pool, therefore being the best reflection of that pool, which is generally referred to as the ovarian reserve. Hence it constitutes the best and most reliable marker for the so called ovarian reserve because primordial follicles are generally metabolically inactive and do not produce markers in large enough quantities that can be measured in peripheral blood. Although we corrected for many potential confounders, residual confounding due to the observation nature of the study might have occurred. Self-reported lifestyle habits such as parental smoking are potential determinants of bisphenols and phthalates. Although we accounted for these in our models, measurements error might have occurred in self-reported lifestyle behaviors.

In conclusion, our study together with previous studies suggest a potential adverse effect of phthalate exposure on the ovary over time. Our results need to be replicated and validated in large longitudinal multi-ethnic population studies. Also, as the formation of primordial follicles takes place in fetal life and bisphenols and phthalates have shown to be able to cross the placental barrier, studying potential transgenerational effects of exposure to endocrine disruptors is of high interest.^29^^,^^34^

## Contributors

SMB: formal analysis, investigation, methodology, and writing—original draft. RD: writing—review and editing. RG: funding acquisition, writing—review and editing. ML: formal analysis, methodology, writing—review and editing. JSL: funding acquisition, writing—review and editing. VWVJ: conceptualization, funding acquisition, project administration, supervision, writing—review and editing. LT: conceptualization, funding acquisition, methodology, supervision, writing—review and editing. SMB and VWVJ accessed and verified the underlying data. All authors read and approved the final version of the manuscript.

## Data sharing statement

The datasets generated and analyzed during the current study are not publicly available due to privacy restrictions but are available from the corresponding author on reasonable request.

## Declaration of interests

SMB is supported by the Royal Netherlands Academy of Arts and Sciences. JSL declares grants from Ferring Pharmaceuticals and Merck, consulting fees from Ferring Pharmaceuticals and Gedeon Richter, honoraria for lectures and support for attending meetings from Ferring Pharmaceuticals, participation to the advisory board of LOCI Trial UK, and is president of the AE PCOS society. LT is supported by the National Institute of Environmental Health Sciences. All other authors declare no conflict of interest.
