# Supplementary material for: Associations of bisphenol and phthalate exposure and anti-Müllerian hormone levels in women of reproductive age
Source: eClinicalMedicine. 2024 Jul 17;74:102734. doi: 10.1016/j.eclinm.2024.102734 (PMC11304696; doi:10.1016/j.eclinm.2024.102734)
Supplement: Supplementary Figs. S1 and S2, Text S1, and Tables S2–S9 [file mmc1.docx]

**Supplementary Material**

**Associations of bisphenol and phthalate exposure and anti-Müllerian hormone levels in women of reproductive age**

Sophia M Blaauwendraad, Ramon HM Dykgraaf, Romy Gaillard, Mengling Liu, Joop S Laven, Vincent WV Jaddoe, Leonardo Trasande

**Figure S1.** Directed Acyclic Graph

**Figure S2.** Correlation plots of the correlation of the bisphenols and phthalates between trimesters.

**Text S1.** Details on chemicals included in grouping per trimester.

**Table S2.** Descriptive statistics of the maternal urinary bisphenol and phthalate metabolites in our study sample (N = 1322)

**Table S3.** Limits of detection, quantification, and percentage of values above limit of detection for our study sample (N = 1322)

**Table S4.** Associations of urinary bisphenol and phthalate concentrations with serum AMH levels in 6 years and 9 years postpartum and on average, basic model.

**Table S5.** Associations of urinary bisphenol and phthalate concentrations with serum AMH levels in 6 years and 9 years postpartum and on average, adjusted model.

**Table S6.** Mediation models for maternal body mass index for the nominal significant associations obtained from linear regression models.

**Table S7.** Associations of urinary bisphenol and phthalate concentrations with serum AMH levels 6 years and 9 years postpartum, and on average among Dutch mothers only (N=744), adjusted model.

**Table S8.** Associations of urinary bisphenol and phthalate concentrations with serum AMH levels obtained from linear mixed effects models, basic model.

**Table S9.** Associations of urinary bisphenol and phthalate concentrations with serum AMH levels obtained from linear mixed effects models, basic model.

**Figure S1. Directed Acyclic Graph**


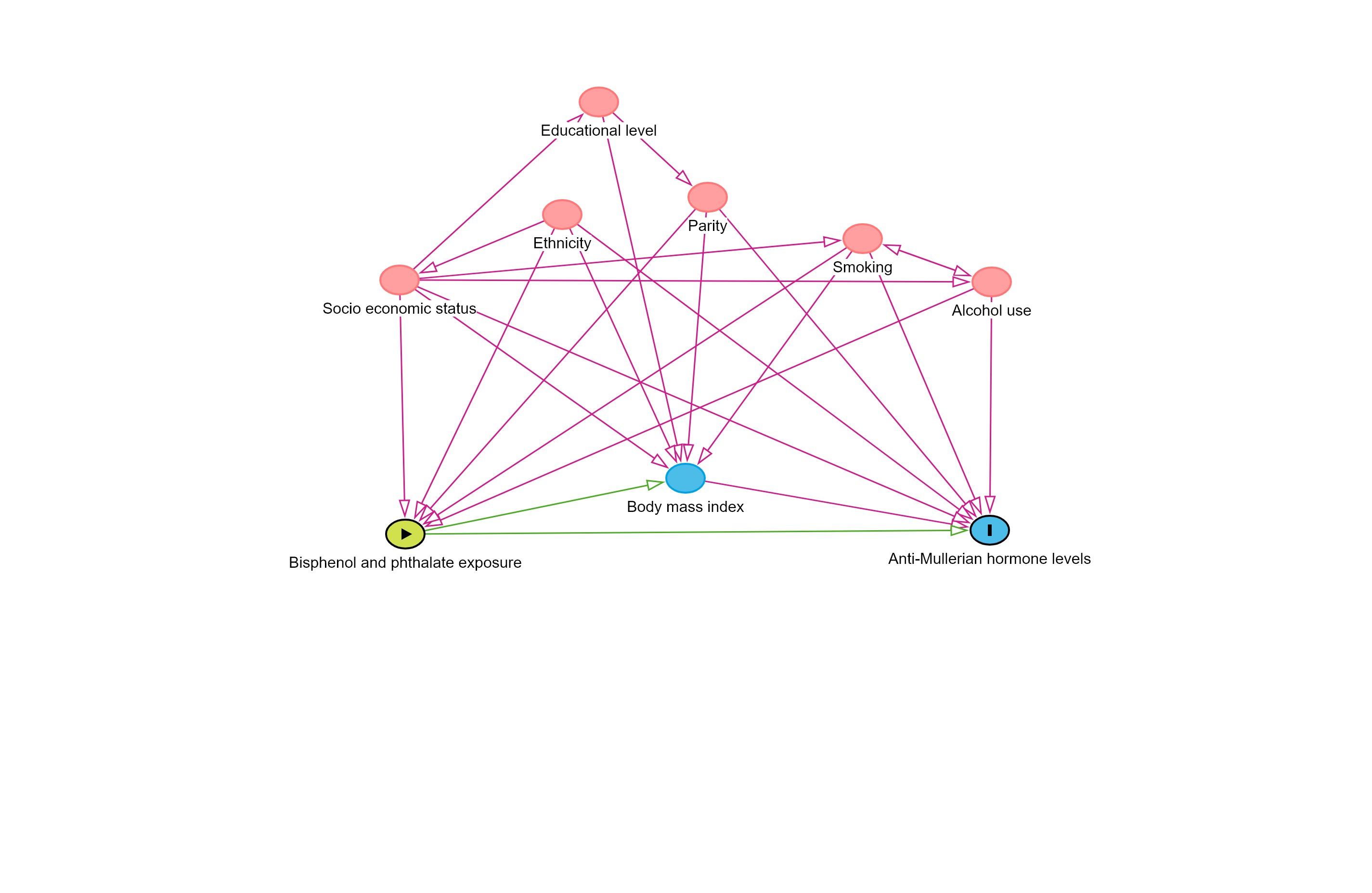


**Figure S2. Correlation plots of the correlation of the bisphenols and phthalates between trimesters.** Values are Spearmans correlation coefficients. BPA bisphenol A, BPS bisphenol S, BPF bisphenol F, PA phthalic acid, mMP monomethylphthalate, mEP monoethylphthalate, mIBP mono-isobutylphthalate, mBP mono-n-butylphthalate, mECPP mono-(2-ethyl-5-carboxypentyl)phthalate, mEHHP mono-(2-ethyl-5-hydroxyhexyl)phthalate, mEOHP mono-(2-ethyl-5-oxohexyl)phthalate, mCMHP mono[(2-carboxymethyl)-hexyl]phthalate, mBzP monobenzylphthalate, mHxP mono-hexylphthalate, mHpP mono-2-heptylphthalate, BP total bisphenol, LMW low molecular weight phthalate, DEHP di-2-ethylhexyl phthalate, DNOP di-n-octylphthalate, HMW high molecular weight phthalate, G1 first trimester, G2 second trimester, G3 third trimester.


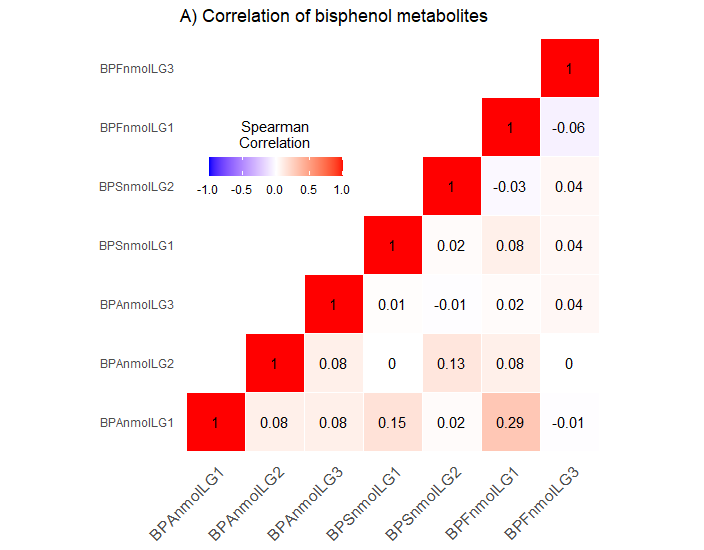

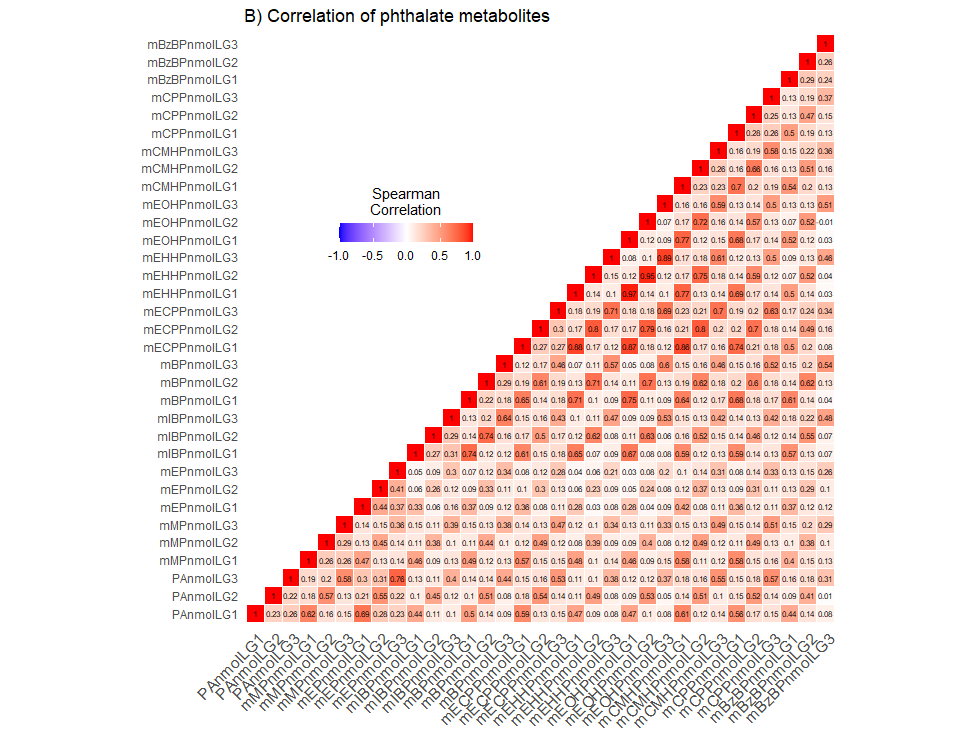


**Text 1. Details on chemicals included in grouping per trimester.**

The low-molecular-weight (LMWP) group in all three trimesters consisted of monomethylphthalate (mMP), monoethylphthalate (mEP), mono-isobutylphthalate (mIBP) and mono-n-butylphthalate (mBP). The high-molecular-weight (HMWP) group in first trimester consisted of the di-2-ethylhexylphthalate (DEHP) group, the di-n-octylphthalate (DNOP) group, (mHxP), mono-2-heptylphthalate (mHpP) and monobenzylphthalate (mBzP). The HMWP group in second and third trimester consisted of the DEHP group, the DNOP group and mBzP. The DEHP group in all three trimesters consisted of mono-(2-ethyl-5-carboxypentyl)phthalate (mECPP), mono-(2-ethyl-5-hydroxyhexyl)phthalate (mEHHP), mono-(2-ethyl-5-oxohexyl)phthalate (mEOHP) and mono[(2-carboxymethyl)-hexyl]phthalate (mCMHP). The DNOP group in all three trimesters consisted of mono(3-carboxypropyl)-phthalate (mCPP).

| **Table S2. Descriptive statistics of the maternal urinary bisphenol and phthalate metabolites in our study sample (N = 1322)** | | | |
| --- | --- | --- | --- |
| **Bisphenol or phthalate metabolite** | **Concentration in ng/mL, median (IQR)** | | |
|  | **Trimester 1** | **Trimester 2** | **Trimester 3** |
| ***Bisphenols*** |  |  |  |
| Bisphenol A (BPA) | 1.13 (0.26, 2.81) | 1.33 (0.62, 2.98) | 1.5 (0.62, 3) |
| Bisphenol S (BPS) | 0.17 (0.03, 0.61) | 0.03 (0.03, 0.1) | 0.03 (0.03, 0.03) |
| Bisphenol F (BPF) | 0.12 (0.12, 0.4) | 0.12 (0.12, 0.12) | 0.12 (0.12, 0.52) |
| Bisphenol Z (BPZ) | 0.09 (0.09, 0.09) | 0.09 (0.09, 0.09) | 0.09 (0.09, 0.09) |
| Bisphenol B (BPB) | 0.02 (0.02, 0.02) | 0.02 (0.02, 0.02) | 0.02 (0.02, 0.02) |
| Bisphenol AP (BPAP) | 0.05 (0.05, 0.05) | 0.05 (0.05, 0.05) | 0.05 (0.05, 0.05) |
| Bisphenol P (BPP) | 0.08 (0.08, 0.08) | 0.08 (0.08, 0.08) | 0.08 (0.08, 0.08) |
| Bisphenol AF (BPAF) | 0.56 (0.56, 0.56) | 0.56 (0.56, 0.56) | 0.56 (0.56, 0.56) |
| ***Phthalic Acid*** | 57.03 (30.6, 121.36) | 153.81 (61.89, 288.56) | 69.5 (33.74, 133.88) |
| ***Low molecular weight phthalates*** |  |  |  |
| Monomethyl phthalate (mMP) | 5.42 (2.75, 9.88) | 3.5 (1.84, 6.3) | 4.09 (2.01, 7.99) |
| Monoethyl phthalate (mEP) | 137.96 (41.22, 486.1) | 73.18 (25.07, 227.34) | 130.85 (44.64, 419.04) |
| Mono-isobutyl phthalate (mIBP) | 21.49 (9.55, 45.72) | 9.05 (4.67, 18.28) | 18.16 (9.36, 38.21) |
| Mono-n-butyl phthalate (mBP) | 16.1 (6.83, 31.09) | 9.75 (5.55, 19.33) | 12.06 (6.15, 25.09) |
| ***High molecular weight phthalates*** |  |  |  |
| ***Di-2-ethylhexyl phthalates*** |  |  |  |
| Mono-(2-ethyl-5-carboxypentyl) phthalate (mECPP) | 16.36 (8.25, 31.78) | 10.61 (5.78, 20.48) | 18.17 (9.53, 33.93) |
| Mono-(2-ethyl-5-hydoxyhexyl phthalate (mEHHP) | 12.02 (5.81, 23.2) | 5.61 (3.01, 10.95) | 10.26 (5.22, 19.91) |
| Mono-(2-ethyl-5-oxohexyl) phthalate (mEOHP) | 7.81 (3.53, 15.48) | 7.54 (3.7, 16.61) | 7.3 (3.85, 14.11) |
| Mono-[(2-carboxymethyl)hexyl] phthalate (mCMHP) | 14.17 (7.6, 26.67) | 4.15 (2.27, 7.4) | 3.48 (1.86, 6.5) |
| ***Di-isononyl phthalate (DINP)*** |  |  |  |
| Monoisononyl phthalate (mINP) | 0.12 (0.12, 0.12) | 0.12 (0.12, 0.12 | 0.12 (0.12, 0.12) |
| ***Di-isodecylphthalate (DIDP)*** |  |  |  |
| Mono-(8-metyl-1-nonyl) phthalate (mIDP) | 0.63 (0.63, 0.63) | 0.63 (0.63, 0.63) | 0.63 (0.63, 0.63) |
| ***Di-n-octylphthalate (DNOP)*** |  |  |  |
| Mono(3-carboxypropyl) phthalate (mCPP) | 1.46 (0.78, 2.78) | 0.89 (0.52, 1.72) | 1.77 (0.95, 3.13) |
| Monooctyl phthalate (mOP) | 0.18 (0.18, 0.18) | 0.18 (0.18, 0.18) | 0.18 (0.18, 0.18) |
| Mono-(7-carboxy-n-heptyl) phthalate (mCHpP) | 0.04 (0.04, 0.04) | 0.04 (0.04, 0.04) | 0.04 (0.04, 0.04) |
| ***Other high molecular weight phthalates*** |  |  |  |
| Monobenzyl phthalate (mBzBP) | 5.78 (2.3, 12.18) | 5.37 (2.23, 11.29) | 3.13 (1.17, 6.46) |
| Mono-hexyl phthalate (mHxP) | 0.22 (0.07, 0.49) | 0.04 (0.04, 0.04) | 0.04 (0.04, 0.04) |
| Mono-2-heptylphthalate (mHpP) | 0.56 (0.21, 1.51) | 0.21 (0.21, 0.21) | 0.21 (0.21, 0.21) |
| Monocyclohexylphthalate (mCHP) | 0.03 (0.03, 0.03) | 0.03 (0.03, 0.03) | 0.03 (0.03, 0.03) |
| Values represent median (interquartile range (IQR)) of the chemicals concentrations in nanogram per milliliter (ng/mL). | | | |

| **Table S3. Limits of detection, quantification and percentage of values above limit of detection for our study sample (N = 1322)** | | | | | |
| --- | --- | --- | --- | --- | --- |
| **Bisphenol or phthalate metabolite** | **Limit of detection (ng/mL)** | **Limit of quantification (ng/mL)** | **% above limit of detection** | | |
|  |  |  | **Trimester 1** | **Trimester 2** | **Trimester 3** |
| ***Bisphenols*** |  |  |  |  |  |
| Bisphenol A (BPA) | 0.15 | 0.50 | 79.1* | 92.9* | 90.2* |
| Bisphenol S (BPS) | 0.05 | 0.15 | 66.5* | 29.9 | 19.4 |
| Bisphenol F (BPF) | 0.18 | 0.59 | 39.8 | 11.5 | 28.9 |
| Bisphenol Z (BPZ) | 0.12 | 0.41 | 12.6 | 4.1 | 0.2 |
| Bisphenol B (BPB) | 0.03 | 0.10 | 9.6 | 2.4 | 0.0 |
| Bisphenol AP (BPAP) | 0.07 | 0.24 | 7.5 | 0.0 | 0.1 |
| Bisphenol P (BPP) | 0.11 | 0.38 | 1.7 | 0.0 | 0.8 |
| Bisphenol AF (BPAF) | 0.79 | 2.61 | 0.0 | 0.0 | 0.1 |
| ***Phthalic Acid*** | 1.11 | 3.67 | 99.7* | 99.9* | 99.5* |
| ***Low molecular weight phthalates*** |  |  |  |  |  |
| Monomethyl phthalate (mMP) | 0.06 | 0.19 | 99.9* | 99.9* | 99.5* |
| Monoethyl phthalate (mEP) | 0.06 | 0.19 | 99.9* | 100.0* | 100.0* |
| Mono-isobutyl phthalate (mIBP) | 0.09 | 0.30 | 99.9* | 100.0* | 99.7* |
| Mono-n-butyl phthalate (mBP) | 0.14 | 0.46 | 99.2* | 100.0* | 99.9* |
| ***High molecular weight phthalates*** |  |  |  |  |  |
| ***Di-2-ethylhexyl phthalates*** |  |  |  |  |  |
| Mono-(2-ethyl-5-carboxypentyl) phthalate (mECPP) | 0.29 | 0.97 | 99.9* | 99.9* | 100.0* |
| Mono-(2-ethyl-5-hydoxyhexyl phthalate (mEHHP) | 0.08 | 0.25 | 99.9* | 99.9* | 99.9* |
| Mono-(2-ethyl-5-oxohexyl) phthalate (mEOHP) | 0.04 | 0.12 | 100.0* | 100.0* | 99.9* |
| Mono-[(2-carboxymethyl)hexyl] phthalate (mCMHP) | 0.04 | 0.13 | 99.9* | 99.8* | 98.9* |
| ***Di-isononyl phthalate (DINP)*** |  |  |  |  |  |
| Monoisononyl phthalate (mINP) | 0.18 | 0.58 | 1.4 | 1.5 | 0.0 |
| ***Di-isodecylphthalate (DIDP)*** |  |  |  |  |  |
| Mono-(8-metyl-1-nonyl) phthalate (mIDP) | 0.89 | 2.93 | 7.5 | 1.9 | 3.3 |
| ***Di-n-octylphthalate (DNOP)*** |  |  |  |  |  |
| Mono(3-carboxypropyl) phthalate (mCPP) | 0.008 | 0.03 | 100.0* | 100.0* | 99.9* |
| Monooctyl phthalate (mOP) | 0.25 | 0.81 | 9.6 | 0.5 | 0.7 |
| Mono-(7-carboxy-n-heptyl) phthalate (mCHpP) | 0.06 | 0.20 | 7.6 | 0.0 | 0.2 |
| ***Other high molecular weight phthalates*** |  |  |  |  |  |
| Monobenzyl phthalate (mBzBP) | 0.15 | 0.50 | 91.6* | 98.2* | 96.5* |
| Mono-hexyl phthalate (mHxP) | 0.06 | 0.19 | 76.0* | 1.2 | 2.0 |
| Mono-2-heptylphthalate (mHpP) | 0.30 | 0.99 | 64.2* | 3.3 | 1.5 |
| Monocyclohexylphthalate (mCHP) | 0.04 | 0.12 | 19.4 | 5.6 | 0.7 |
| *>50% of samples above limit of detection | | | |  |  |

| **Table S4. Associations of urinary bisphenol and phthalate concentrations with serum AMH levels in 6 years and 9 years postpartum, on average and change in AMH, basic model.** | | | | | | | | | | | | |
| --- | --- | --- | --- | --- | --- | --- | --- | --- | --- | --- | --- | --- |
| **Bisphenol or phthalate metabolite (group)** | **6 years postpartum AMH** | | | **9 years postpartum AMH** | | | **Average AMH** | | | **Change in AMH** | | |
|  | **Estimate** | **Nominal p-value** | **FDR p-value** | **Estimate** | **Nominal p-value** | **FDR P-value** | **Estimate** | **Nominal p-value** | **FDR P-value** | **Estimate** | **Nominal p-value** | **FDR P-value** |
| Bisphenol A (BPA) | 0.03 (-0.02, 0.08) | 0.18 | 0.28 | -0.03 (-0.1, 0.04) | 0.37 | 0.420 | 0.00 (-0.05, 0.05) | 0.91 | 0.98 | -0.03 (-0.10, 0.04) | 0.45 | 0.66 |
| ***Phthalic Acid*** | -0.02 (-0.08, 0.04) | 0.46 | 0.49 | -0.07 (-0.15, 0.02) | 0.11 | 0.160 | -0.04 (-0.10, 0.02) | 0.21 | 0.25 | -0.04 (-0.13, 0.04) | 0.32 | 0.59 |
| ***Low molecular weight phthalate*** | -0.02 (-0.06, 0.02) | 0.39 | 0.45 | -0.02 (-0.08, 0.04) | 0.55 | 0.581 | -0.02 (-0.06, 0.03) | 0.44 | 0.51 | 0.00 (-0.06, 0.06) | 0.93 | 0.93 |
| Monomethyl phthalate (mMP) | -0.03 (-0.08, 0.03) | 0.32 | 0.40 | -0.05 (-0.12, 0.02) | 0.19 | 0.234 | -0.04 (-0.09, 0.01) | 0.12 | 0.20 | -0.02 (-0.09, 0.05) | 0.55 | 0.73 |
| Monoethyl phthalate (mEP) | 0.00 (-0.04, 0.03) | 0.94 | 0.94 | 0 .00 (-0.04, 0.05) | 0.88 | 0.880 | 0.00 (-0.04, 0.04) | 0.98 | 0.98 | 0.00 (-0.06, 0.05) | 0.86 | 0.92 |
| Mono-isobutyl phthalate (mIBP) | -0.10 (-0.15, -0.05)* | 0.00013 | 0.0021 | -0.11 (-0.18, -0.04)* | 0.0019 | 0.015 | -0.08 (-0.13, -0.03)* | 0.0018 | 0.015 | -0.01 (-0.09, 0.06) | 0.77 | 0.88 |
| Mono-n-butyl phthalate (mBP) | -0.06 (-0.12, -0.01) | 0.029 | 0.060 | -0.10 (-0.18, -0.02)* | 0.016 | 0.041 | -0.07 (-0.13, -0.02)* | 0.013 | 0.042 | -0.05 (-0.13, 0.03) | 0.25 | 0.59 |
| ***High molecular weight phthalate*** | -0.07 (-0.13, -0.01) | 0.029 | 0.060 | -0.1 0 (-0.18, -0.01) | 0.030 | 0.059 | -0.07 (-0.13, 0.00) | 0.035 | 0.078 | -0.05 (-0.14, 0.04) | 0.26 | 0.59 |
| ***Di-2-ethylhexyl phthalates*** | -0.07 (-0.13, -0.01) | 0.030 | 0.060 | -0.09 (-0.17, 0.00) | 0.039 | 0.069 | -0.07 (-0.13, 0.00) | 0.038 | 0.078 | -0.05 (-0.14, 0.04) | 0.25 | 0.59 |
| Mono-(2-ethyl-5-carboxypentyl) phthalate (mECPP) | -0.07 (-0.12, -0.01) | 0.030 | 0.060 | -0.07 (-0.15, 0.01) | 0.079 | 0.126 | -0.06 (-0.12, 0.00) | 0.039 | 0.078 | -0.02 (-0.10, 0.07) | 0.68 | 0.83 |
| Mono-(2-ethyl-5-hydoxyhexyl phthalate (mEHHP) | -0.08 (-0.14, -0.02)* | 0.0084 | 0.034 | -0.10 (-0.18, -0.02)* | 0.021 | 0.048 | -0.09 (-0.15, -0.03)* | 0.0051 | 0.025 | -0.06 (-0.15, 0.03) | 0.16 | 0.59 |
| Mono-(2-ethyl-5-oxohexyl) phthalate (mEOHP) | -0.08 (-0.14, -0.02)* | 0.0060 | 0.032 | -0.11 (-0.19, -0.02)* | 0.011 | 0.035 | -0.08 (-0.14, -0.02)* | 0.0063 | 0.025 | -0.05 (-0.14, 0.03) | 0.22 | 0.59 |
| Mono-[(2-carboxymethyl)hexyl] phthalate (mCMHP) | -0.04 (-0.10, 0.02) | 0.16 | 0.28 | -0.07 (-0.15, 0.02) | 0.12 | 0.160 | -0.05 (-0.11, 0.01) | 0.10 | 0.15 | -0.06 (-0.14, 0.03) | 0.18 | 0.59 |
| ***Di-n-octylphthalate (DNOP)*** | -0.04 (-0.11, 0.02) | 0.21 | 0.28 | -0.12 (-0.21, -0.04)* | 0.0062 | 0.025 | -0.06 (-0.13, 0.00) | 0.057 | 0.091 | -0.05 (-0.14, 0.05) | 0.33 | 0.59 |
| Mono(3-carboxypropyl) phthalate (mCPP) | -0.04 (-0.11, 0.02) | 0.21 | 0.28 | -0.12 (-0.21, -0.04)* | 0.0062 | 0.025 | -0.06 (-0.13, 0.00) | 0.057 | 0.091 | -0.05 (-0.14, 0.05) | 0.33 | 0.59 |
| Monobenzyl phthalate (mBzBP) | -0.08 (-0.13, -0.03)* | 0.0024 | 0.019 | -0.12 (-0.19, -0.05)* | 0.00059 | 0.009 | -0.09 (-0.14, -0.04)* | 0.00062 | 0.010 | -0.03 (-0.10, 0.04) | 0.45 | 0.66 |
| Models reflect the change in maternal serum Antimüllerian (AMH) hormone levels (ug/L) at 6 years and 9 years postpartum, on average, and on change in AMH between 6 and 9 years postpartum per doubling in maternal urinary pregnancy-averaged bisphenol or phthalate metabolite or metabolite group (nmol/L). Models are adjusted for maternal urinary creatinine concentrations and age. *False Discovery Rate (FDR) adjusted p-value <0.05 | | | | | | | | | | | | |

| **Table S5. Associations of urinary bisphenol and phthalate concentrations with serum AMH levels 6 years and 9 years postpartum, and on average, adjusted model.** | | | | | | | | | | | | |
| --- | --- | --- | --- | --- | --- | --- | --- | --- | --- | --- | --- | --- |
| **Bisphenol or phthalate metabolite (group)** | **6 years postpartum AMH** | | | **9 years postpartum AMH** | | | **Average AMH** | | | **Change in AMH** | | |
|  | **Estimate** | **Nominal p-value** | **FDR P-value** | **Estimate** | **Nominal p-value** | **FDR P-value** | **Estimate** | **Nominal p-value** | **FDR P-value** | **Estimate** | **Nominal p-value** | **FDR P-value** |
| Bisphenol A (BPA) | 0.04 (-0.01, 0.09) | 0.11 | 0.20 | -0.01 (-0.08, 0.06) | 0.72 | 0.76 | 0.01 (-0.04, 0.06) | 0.74 | 0.79 | -0.03 (-0.10, 0.05) | 0.46 | 0.73 |
| ***Phthalic Acid*** | -0.01 (-0.07, 0.06) | 0.81 | 0.80 | -0.04 (-0.12, 0.04) | 0.35 | 0.42 | -0.02 (-0.09, 0.04) | 0.47 | 0.58 | -0.04 (-0.13, 0.05) | 0.41 | 0.73 |
| ***Low molecular weight phthalate*** | -0.01 (-0.05, 0.04) | 0.75 | 0.80 | 0.01 (-0.05, 0.07) | 0.85 | 0.85 | 0 .00 (-0.05, 0.04) | 0.87 | 0.87 | 0.00 (-0.06, 0.07) | 0.89 | 0.98 |
| Monomethyl phthalate (mMP) | -0.02 (-0.07, 0.03) | 0.38 | 0.47 | -0.05 (-0.11, 0.02) | 0.20 | 0.29 | -0.04 (-0.09, 0.02) | 0.17 | 0.25 | -0.02 (-0.09, 0.06) | 0.68 | 0.91 |
| Monoethyl phthalate (mEP) | 0.01 (-0.03, 0.04) | 0.75 | 0.80 | 0.02 (-0.03, 0.07) | 0.40 | 0.46 | 0.01 (-0.03, 0.05) | 0.62 | 0.71 | 0.00 (-0.05, 0.05) | 0.98 | 0.98 |
| Mono-isobutyl phthalate (mIBP) | -0.08 (-0.13, -0.03)* | 0.0028 | 0.045 | -0.08 (-0.16, -0.01) | 0.029 | 0.093 | -0.07 (-0.12, -0.01) | 0.015 | 0.061 | 0.00 (-0.08, 0.08) | 0.98 | 0.98 |
| Mono-n-butyl phthalate (mBP) | -0.05 (-0.11, 0.01) | 0.10 | 0.20 | -0.08 (-0.16, 0.01) | 0.068 | 0.16 | -0.06 (-0.12, 0.00) | 0.041 | 0.12 | -0.04 (-0.13, 0.04) | 0.35 | 0.69 |
| ***High molecular weight phthalate*** | -0.06 (-0.12, 0.00) | 0.062 | 0.15 | -0.08 (-0.16, 0.01) | 0.079 | 0.16 | -0.06 (-0.12, 0.01) | 0.072 | 0.12 | -0.05 (-0.14, 0.05) | 0.32 | 0.69 |
| ***Di-2-ethylhexyl phthalates*** | -0.06 (-0.12, 0.00) | 0.064 | 0.15 | -0.07 (-0.16, 0.01) | 0.096 | 0.17 | -0.06 (-0.12, 0.01) | 0.077 | 0.12 | -0.05 (-0.13, 0.04) | 0.32 | 0.69 |
| Mono-(2-ethyl-5-carboxypentyl) phthalate (mECPP) | -0.06 (-0.12, 0.00) | 0.063 | 0.15 | -0.06 (-0.14, 0.02) | 0.15 | 0.24 | -0.05 (-0.11, 0.01) | 0.077 | 0.12 | -0.01 (-0.10, 0.07) | 0.77 | 0.95 |
| Mono-(2-ethyl-5-hydoxyhexyl phthalate (mEHHP) | -0.08 (-0.14, -0.01) | 0.018 | 0.073 | -0.08 (-0.17, 0.00) | 0.052 | 0.14 | -0.08 (-0.15, -0.02) | 0.010 | 0.061 | -0.06 (-0.15, 0.03) | 0.22 | 0.69 |
| Mono-(2-ethyl-5-oxohexyl) phthalate (mEOHP) | -0.08 (-0.13, -0.02) | 0.012 | 0.073 | -0.09 (-0.18, -0.01) | 0.025 | 0.093 | -0.08 (-0.14, -0.02) | 0.011 | 0.061 | -0.05 (-0.13, 0.04) | 0.29 | 0.69 |
| Mono-[(2-carboxymethyl)hexyl] phthalate (mCMHP) | -0.03 (-0.09, 0.03) | 0.29 | 0.39 | -0.05 (-0.13, 0.04) | 0.27 | 0.36 | -0.04 (-0.1, 0.02) | 0.21 | 0.28 | -0.05 (-0.14, 0.03) | 0.23 | 0.69 |
| ***Di-n-octylphthalate (DNOP)*** | -0.04 (-0.10, 0.03) | 0.24 | 0.35 | -0.11 (-0.20, -0.03) | 0.011 | 0.076 | -0.06 (-0.13, 0.00) | 0.068 | 0.12 | -0.04 (-0.14, 0.05) | 0.34 | 0.69 |
| Mono(3-carboxypropyl) phthalate (mCPP) | -0.04 (-0.10, 0.03) | 0.24 | 0.35 | -0.11 (-0.20, -0.03) | 0.011 | 0.076 | -0.06 (-0.13, 0.00) | 0.068 | 0.12 | -0.04 (-0.14, 0.05) | 0.34 | 0.69 |
| Monobenzyl phthalate (mBzBP) | -0.06 (-0.11, -0.01) | 0.018 | 0.073 | -0.09 (-0.16, -0.02) | 0.014 | 0.076 | -0.07 (-0.13, -0.02) | 0.0049 | 0.061 | -0.02 (-0.10, 0.06) | 0.61 | 0.89 |
| Models reflect the change in maternal serum Antimüllerian hormone (AMH) levels (ug/L) at 6 years and 9 years postpartum, on average, and on change in AMH between 6 and 9 years postpartum per doubling in maternal urinary pregnancy-averaged bisphenol or phthalate metabolite or metabolite group (nmol/L). Models are adjusted for maternal urinary creatinine concentrations. maternal age at outcome measurement, ethnicity, educational level, parity, smoking, alcohol use and body-mass index at exposure measurement. *False Discovery Rate (FDR) adjusted p-value <0.05. | | | | | | | | | | | | |

| **Table S6. Mediation models for maternal body mass index for the nominal significant associations obtained from linear regression models.** | | | | | | | |
| --- | --- | --- | --- | --- | --- | --- | --- |
| **Outcome** | **Exposure** | **Average causal mediation effect ^a^** | | **Average direct effect ^b^** | | **Proportion Mediated** | |
|  |  | **Estimate (95% CI)** | **P-value** | **Estimate (95% CI)** | **P-value** | **Estimate (95% CI)** | **P-value** |
| AMH 6 years | mIBP | 0.00 (-0.01, 0.00) | 0.38 | -0.08 (-0.13, -0.02) | <0.0001 | 0.03 (-0.05, 0.22) | 0.38 |
|  | mEHHP | 0.00 (-0.01, 0.00) | 0.28 | -0.07 (-0.14, -0.01) | 0.028 | 0.03 (-0.03, 0.20) | 0.30 |
|  | mEOHP | 0.00 (-0.01, 0.00) | 0.28 | -0.07 (-0.13, -0.01) | 0.032 | 0.03 (-0.04, 0.23) | 0.30 |
|  | mBzBP | 0.00 (-0.01, 0.00) | 0.31 | -0.06 (-0.11, -0.01) | 0.030 | 0.04 (-0.07, 0.28) | 0.32 |
| AMH 9 years | mIBP | -0.01 (-0.01, 0.00) | 0.14 | -0.08 (-0.15, 0.00) | 0.042 | 0.06 (-0.05, 0.36) | 0.16 |
|  | mEOHP | 0.00 (-0.01, 0.00) | 0.25 | -0.09 (-0.16, 0.00) | 0.052 | 0.03 (-0.03, 0.24) | 0.27 |
|  | mCPP | -0.01 (-0.02, 0.00) | 0.15 | -0.11 (-0.21, -0.01) | 0.022 | 0.04 (-0.03, 0.26) | 0.16 |
|  | mBzBP | -0.00 (-0.01, 0.00) | 0.15 | -0.08 (-0.15, -0.02) | 0.016 | 0.05 (-0.02, 0.26) | 0.16 |
| ^a^ Values represent regression coefficients (95% confidence interval (CI)) of the regression models that reflect the difference in standard deviation score (SDS) of maternal body-mass index (BMI) at outcome measurement per doubling in urinary phthalate and bisphenol (nmol/L). Models are adjusted for maternal urinary creatinine concentrations. maternal age at outcome measurement, ethnicity, educational level, parity, smoking and alcohol use.  ^b^ Values represent regression coefficients (95% confidence interval (CI)) of the regression models that reflect the change in maternal serum Antimüllerian hormone (AMH) levels (ug/L) per doubling in urinary phthalate and bisphenol (nmol/L). Models are adjusted for maternal urinary creatinine concentrations. maternal age at outcome measurement, ethnicity, educational level, parity, smoking and alcohol use.  ^c^ Values represent regression coefficients (95% confidence interval (CI)) of the regression models that reflect the change in maternal serum Antimüllerian hormone (AMH) levels (ug/L) per doubling in urinary phthalate and bisphenol (nmol/L). Models are adjusted for maternal urinary creatinine concentrations. maternal age at outcome measurement, ethnicity, educational level, parity, smoking, alcohol use, and BMI at outcome measurement. | | | | | | | |

| **Table S7. Associations of urinary bisphenol and phthalate concentrations with serum AMH levels 6 years and 9 years postpartum, and on average among Dutch mothers only (N=744), adjusted model** | | | | | | | | | | | | |
| --- | --- | --- | --- | --- | --- | --- | --- | --- | --- | --- | --- | --- |
| **Bisphenol or phthalate metabolite (group)** | **6 years postpartum AMH** | | | **9 years postpartum AMH** | | | **Average AMH** | | | **Change in AMH** | | |
|  | **Estimate** | **Nominal p-value** | **FDR P-value** | **Estimate** | **Nominal p-value** | **FDR P-value** | **Estimate** | **Nominal p-value** | **FDR P-value** | **Estimate** | **Nominal p-value** | **FDR P-value** |
| Bisphenol A (BPA) | 0.00 (-0.07, 0.07) | 0.99 | 0.99 | -0.02 (-0.12, 0.07) | 0.63 | 0.82 | -0.04 (-0.11, 0.03) | 0.27 | 0.90 | 0.02 (-0.06, 0.11) | 0.58 | 0.91 |
| ***Phthalic Acid*** | 0.02 (-0.06, 0.10) | 0.64 | 0.98 | 0.03 (-0.08, 0.13) | 0.62 | 0.82 | 0.01 (-0.07, 0.10) | 0.79 | 0.90 | -0.10 (-0.2, 0.00) | 0.046 | 0.72 |
| ***Low molecular weight phthalate*** | 0.02 (-0.04, 0.08) | 0.50 | 0.98 | 0.05 (-0.02, 0.13) | 0.18 | 0.79 | 0.03 (-0.03, 0.09) | 0.33 | 0.90 | -0.02 (-0.10, 0.05) | 0.546 | 0.91 |
| Monomethyl phthalate (mMP) | -0.04 (-0.11, 0.03) | 0.29 | 0.98 | -0.04 (-0.14, 0.05) | 0.34 | 0.79 | -0.05 (-0.13, 0.02) | 0.16 | 0.90 | -0.04 (-0.12, 0.05) | 0.41 | 0.91 |
| Monoethyl phthalate (mEP) | 0.02 (-0.03, 0.07) | 0.38 | 0.98 | 0.05 (-0.01, 0.11) | 0.11 | 0.79 | 0.03 (-0.02, 0.08) | 0.19 | 0.90 | -0.02 (-0.08, 0.04) | 0.59 | 0.91 |
| Mono-isobutyl phthalate (mIBP) | -0.01 (-0.09, 0.07) | 0.83 | 0.98 | -0.04 (-0.15, 0.06) | 0.40 | 0.79 | -0.01 (-0.09, 0.07) | 0.80 | 0.90 | -0.02 (-0.12, 0.08) | 0.67 | 0.91 |
| Mono-n-butyl phthalate (mBP) | 0.00 (-0.08, 0.09) | 0.95 | 1.00 | 0.01 (-0.1, 0.12) | 0.82 | 0.82 | -0.02 (-0.11, 0.06) | 0.57 | 0.90 | -0.04 (-0.14, 0.07) | 0.49 | 0.91 |
| ***High molecular weight phthalate*** | -0.02 (-0.11, 0.08) | 0.73 | 0.98 | -0.03 (-0.15, 0.09) | 0.68 | 0.82 | -0.01 (-0.11, 0.09) | 0.84 | 0.90 | -0.02 (-0.13, 0.10) | 0.76 | 0.91 |
| ***Di-2-ethylhexyl phthalates*** | -0.02 (-0.11, 0.07) | 0.73 | 0.98 | -0.02 (-0.14, 0.1) | 0.72 | 0.82 | -0.01 (-0.10, 0.09) | 0.85 | 0.90 | -0.02 (-0.13, 0.09) | 0.75 | 0.91 |
| Mono-(2-ethyl-5-carboxypentyl) phthalate (mECPP) | -0.01 (-0.1, 0.07) | 0.78 | 0.98 | -0.01 (-0.12, 0.09) | 0.82 | 0.82 | 0.00 (-0.08, 0.09) | 0.94 | 0.94 | -0.01 (-0.11, 0.09) | 0.85 | 0.91 |
| Mono-(2-ethyl-5-hydoxyhexyl phthalate (mEHHP) | -0.03 (-0.13, 0.06) | 0.47 | 0.98 | -0.05 (-0.17, 0.07) | 0.39 | 0.79 | -0.04 (-0.13, 0.06) | 0.43 | 0.90 | -0.01 (-0.12, 0.10) | 0.86 | 0.91 |
| Mono-(2-ethyl-5-oxohexyl) phthalate (mEOHP) | -0.03 (-0.12, 0.06) | 0.56 | 0.98 | -0.04 (-0.15, 0.08) | 0.51 | 0.82 | -0.03 (-0.12, 0.06) | 0.53 | 0.90 | -0.03 (-0.14, 0.08) | 0.61 | 0.91 |
| Mono-[(2-carboxymethyl)hexyl] phthalate (mCMHP) | -0.03 (-0.12, 0.06) | 0.54 | 0.98 | -0.03 (-0.15, 0.09) | 0.67 | 0.82 | -0.04 (-0.13, 0.05) | 0.41 | 0.90 | 0.02 (-0.1, 0.14) | 0.74 | 0.91 |
| ***Di-n-octylphthalate (DNOP)*** | 0.01 (-0.08, 0.10) | 0.86 | 0.98 | -0.07 (-0.19, 0.05) | 0.22 | 0.79 | -0.02 (-0.11, 0.08) | 0.71 | 0.90 | -0.03 (-0.14, 0.08) | 0.62 | 0.91 |
| Mono(3-carboxypropyl) phthalate (mCPP) | 0.01 (-0.08, 0.10) | 0.86 | 0.98 | -0.07 (-0.19, 0.05) | 0.22 | 0.79 | -0.02 (-0.11, 0.08) | 0.71 | 0.90 | -0.03 (-0.14, 0.08) | 0.62 | 0.91 |
| Monobenzyl phthalate (mBzBP) | -0.02 (-0.09, 0.05) | 0.60 | 0.98 | -0.04 (-0.14, 0.05) | 0.38 | 0.79 | -0.03 (-0.11, 0.04) | 0.38 | 0.90 | 0.01 (-0.09, 0.10) | 0.91 | 0.91 |
| Models reflect the change in maternal serum Antimüllerian hormone (AMH) levels (ug/L) at 6 years and 9 years postpartum, on average, and on change in AMH between 6 and 9 years postpartum per doubling in maternal urinary pregnancy-averaged bisphenol or phthalate metabolite or metabolite group (nmol/L). Models are adjusted for maternal urinary creatinine concentrations, maternal age at outcome measurement, ethnicity, educational level, parity, smoking, alcohol use and body-mass index at exposure measurement. | | | | | | | | | | | | |

| **Table S8. Associations of urinary bisphenol and phthalate concentrations with serum AMH levels obtained from linear mixed effects models, basic model.** | | | |
| --- | --- | --- | --- |
| **Bisphenol or phthalate metabolite (group)** | **AMH** | | |
|  | **Estimate** | **Nominal p-value** | **FDR P-value** |
| Bisphenol A (BPA) | 0.00 (-0.05, 0.05) | 0.92 | 0.92 |
| ***Phthalic Acid*** | -0.04 (-0.11, 0.02) | 0.19 | 0.25 |
| ***Low molecular weight phthalate*** | -0.02 (-0.07, 0.02) | 0.36 | 0.42 |
| Monomethyl phthalate (mMP) | -0.03 (-0.09, 0.02) | 0.21 | 0.26 |
| Monoethyl phthalate (mEP) | 0.00 (-0.04, 0.04) | 0.92 | 0.92 |
| Mono-isobutyl phthalate (mIBP) | -0.10 (-0.15, -0.04)* | 0.00033 | 0.0026 |
| Mono-n-butyl phthalate (mBP) | -0.08 (-0.14, -0.02)* | 0.012 | 0.040 |
| ***High molecular weight phthalate*** | -0.07 (-0.14, -0.01) | 0.030 | 0.062 |
| ***Di-2-ethylhexyl phthalates*** | -0.07 (-0.13, 0.00) | 0.036 | 0.063 |
| Mono-(2-ethyl-5-carboxypentyl) phthalate (mECPP) | -0.06 (-0.12, 0.00) | 0.059 | 0.094 |
| Mono-(2-ethyl-5-hydoxyhexyl phthalate (mEHHP) | -0.10 (-0.16, -0.03)* | 0.0034 | 0.018 |
| Mono-(2-ethyl-5-oxohexyl) phthalate (mEOHP) | -0.09 (-0.15, -0.03)* | 0.0047 | 0.019 |
| Mono-[(2-carboxymethyl)hexyl] phthalate (mCMHP) | -0.06 (-0.12, 0.00) | 0.065 | 0.095 |
| ***Di-n-octylphthalate (DNOP)*** | -0.07 (-0.14, -0.01) | 0.031 | 0.062 |
| Mono(3-carboxypropyl) phthalate (mCPP) | -0.07 (-0.14, -0.01) | 0.031 | 0.062 |
| Monobenzyl phthalate (mBzBP) | -0.10 (-0.15, -0.04)* | 0.00026 | 0.0026 |
| Models reflect the change in maternal serum Antimüllerian (AMH) hormone levels (ug/L) per doubling in maternal urinary pregnancy-averaged bisphenol or phthalate metabolite or metabolite group (nmol/L). Effect estimates were obtained from linear mixed-effects models and adjusted for maternal urinary creatinine concentrations and age at baseline. *False Discovery Rate (FDR) adjusted p-value <0.05. | | | |

| **Table S9. Associations of urinary bisphenol and phthalate concentrations with serum AMH levels obtained from linear mixed effects models, adjusted model.** | | | |
| --- | --- | --- | --- |
| **Bisphenol or phthalate metabolite (group)** | **AMH** | | |
|  | **Estimate** | **Nominal p-value** | **FDR P-value** |
| Bisphenol A (BPA) | 0.00 (-0.05, 0.06) | 0.92 | 0.92 |
| ***Phthalic Acid*** | -0.03 (-0.09, 0.04) | 0.45 | 0.55 |
| ***Low molecular weight phthalate*** | -0.01 (-0.05, 0.04) | 0.81 | 0.87 |
| Monomethyl phthalate (mMP) | -0.03 (-0.09, 0.02) | 0.26 | 0.34 |
| Monoethyl phthalate (mEP) | 0.01 (-0.03, 0.05) | 0.70 | 0.80 |
| Mono-isobutyl phthalate (mIBP) | -0.07 (-0.13, -0.02)* | 0.0093 | 0.037 |
| Mono-n-butyl phthalate (mBP) | -0.06 (-0.12, 0.00) | 0.061 | 0.13 |
| ***High molecular weight phthalate*** | -0.06 (-0.13, 0.00) | 0.065 | 0.13 |
| ***Di-2-ethylhexyl phthalates*** | -0.06 (-0.13, 0.01) | 0.072 | 0.13 |
| Mono-(2-ethyl-5-carboxypentyl) phthalate (mECPP) | -0.06 (-0.12, 0.01) | 0.085 | 0.14 |
| Mono-(2-ethyl-5-hydoxyhexyl phthalate (mEHHP) | -0.09 (-0.15, -0.02)* | 0.0074 | 0.037 |
| Mono-(2-ethyl-5-oxohexyl) phthalate (mEOHP) | -0.08 (-0.14, -0.02)* | 0.0093 | 0.037 |
| Mono-[(2-carboxymethyl)hexyl] phthalate (mCMHP) | -0.04 (-0.11, 0.02) | 0.17 | 0.24 |
| ***Di-n-octylphthalate (DNOP)*** | -0.07 (-0.14, 0.00) | 0.045 | 0.12 |
| Mono(3-carboxypropyl) phthalate (mCPP) | -0.07 (-0.14, 0.00) | 0.045 | 0.12 |
| Monobenzyl phthalate (mBzBP) | -0.08 (-0.13, -0.03)* | 0.0039 | 0.037 |
| Models reflect the change in maternal serum Antimüllerian (AMH) hormone levels (ug/L) per doubling in maternal urinary pregnancy-averaged bisphenol or phthalate metabolite or metabolite group (nmol/L). Effect estimates were obtained from linear mixed-effects models and adjusted for maternal urinary creatinine concentrations, maternal age at baseline, ethnicity, educational level, parity, smoking, alcohol use and body-mass index at exposure measurement. *False Discovery Rate (FDR) adjusted p-value <0.05. | | | |
